# Unmasked immune reconstitution inflammatory syndrome towards B-cell non-Hodgkin lymphoma during treatment of esophageal actinomycosis in a patient with advanced HIV: a case report

**DOI:** 10.1186/s12981-023-00526-y

**Published:** 2023-07-14

**Authors:** Elsa K. Vargas-Garcia, Augusto R. Fernandez-Aristi, Gonzalo Cornejo-Venegas, Juan José Montenegro-Idrogo, Juan Chirinos-Vega, Alfredo Chiappe-Gonzalez

**Affiliations:** 1 Universidad Peruana de Ciencias Aplicadas, Avenida Alameda San Marcos cuadra 2 S/N, Chorrillos, Lima, 15023 Peru; 2 Hospital Nacional Dos de Mayo. Parque “Historia de la Medicina Peruana” S/N, Lima, 15003 Peru; 3 Facultad de Ciencias de la Salud, Universidad Científica del Sur. Carretera Panamericana, Sur km19, Villa El Salvador, Lima, 15067 Peru; 4 Centro de Investigaciones Tecnológicas, Biomédicas y Medioambientales, Universidad Nacional Mayor de San Marcos, Lima, 070102 Peru; 5 Instituto de Investigaciones en Ciencias Biomédicas, Universidad Ricardo Palma, Lima, Peru; 6 Clínica Angloamericana, Calle Alfredo Salazar 350, Lima, San Isidro 15073 Peru

**Keywords:** Human immunodeficiency virus, Actinomycosis, Immune reconstitution inflammatory syndrome, Non-Hodgkin lymphoma

## Abstract

**Background:**

Actinomycosis is an unusual chronic bacterial infection, even rarer in people living with HIV. It is not considered an AIDS-defining disease. However, the role in co-presentation or overlap with other opportunistic conditions of advanced HIV is unknown.

**Case presentation:**

A 49-year-old Peruvian male presented with a 4-month history of dysphagia, odynophagia, hyporexia and wasting. He underwent an upper digestive endoscopy, in which ulcers with a necrotic center were observed, therefore, the initial diagnostic assumption was esophageal cancer. Subsequent pathology report excluded neoplasms and confirmed the diagnosis of actinomycosis. Serology for human immunodeficiency virus was requested, yielding a positive result. Antimicrobial treatment with amoxicillin and antiretroviral therapy were indicated, with slow clinical improvement. After 4 months, epigastric discomfort presented, for which a new upper digestive endoscopy was performed, revealing a deep gastric ulcer, which was compatible with diffuse large B-cell non-Hodgkin lymphoma.

**Conclusion:**

Esophageal actinomycosis in people living with HIV is very rare. We suggest HIV-associated immunosuppression is not enough to allow for actinomycosis to develop, and masked underlying entities should be sought. The existence of such entities in people living with HIV should raise awareness of the possibility of unmasked immune reconstitution inflammatory syndrome once treatment has started.

## Introduction

Actinomycosis is a rare subacute-to-chronic infection caused by gram-positive, non-spore forming, non-acid fast, facultative-to-strictly-anaerobic bacilliform bacteria from the genus *Actinomyces* spp. *Actinomyces israelii* is the species that most frequently causes human infections. [1] Because it is an uncommon disease, there are no accurate reports of its incidence, with some series suggesting most large centers may see up to one case per year. [2]

*Actinomyces* spp. are part of normal flora of human mucosae in the gastrointestinal, respiratory, and genitourinary tract; and only causes disease when there is mucosal barrier breach. As such, common risk factors for actinomycosis include dental and periodontal disease; as well as immunosuppressive states including diabetes mellitus, AIDS and primary immunodeficiencies in children. [3]

Actinomycosis is not a disease usually associated with HIV. However, a brief literature review found esophageal, [4, 5] respiratory, [6] and cutaneous [7] actinomycosis reports in people living with HIV (PLWH). Usually, these reports describe an underlying inflammatory process was present at the time of infection, such as esophageal candidiasis or cytomegalovirus esophagitis.

We report an unusual case of esophageal actinomycosis in a PLWH, that developed a mucosal-associated lymphoid tissue (MALT) lymphoma-associated immune reconstitution inflammatory syndrome (IRIS) after antiretroviral treatment was started.

## Case presentation

A 49-year-old male, from Callao, Peru, with a history of untreated arterial hypertension, COVID-19, and bacterial pneumonia two years prior to the current illness presented to the infectious diseases consult with a 4-month history of dysphagia to solids and later to liquids, odynophagia, hyporexia and significant weight loss (22 kg). The physical examination highlighted normal vital functions, a considerable decrease in subcutaneous cell tissue, onychodystrophy of the feet, scaly erythema on the scalp, and enlarged cervical lymph nodes (the largest at 12 mm). Blood biochemistry showed moderate anemia (Hb 8.1 g/dl) and severe lymphopenia (total lymphocyte count 434 cells/mm^3^).

**Fig. 1:**
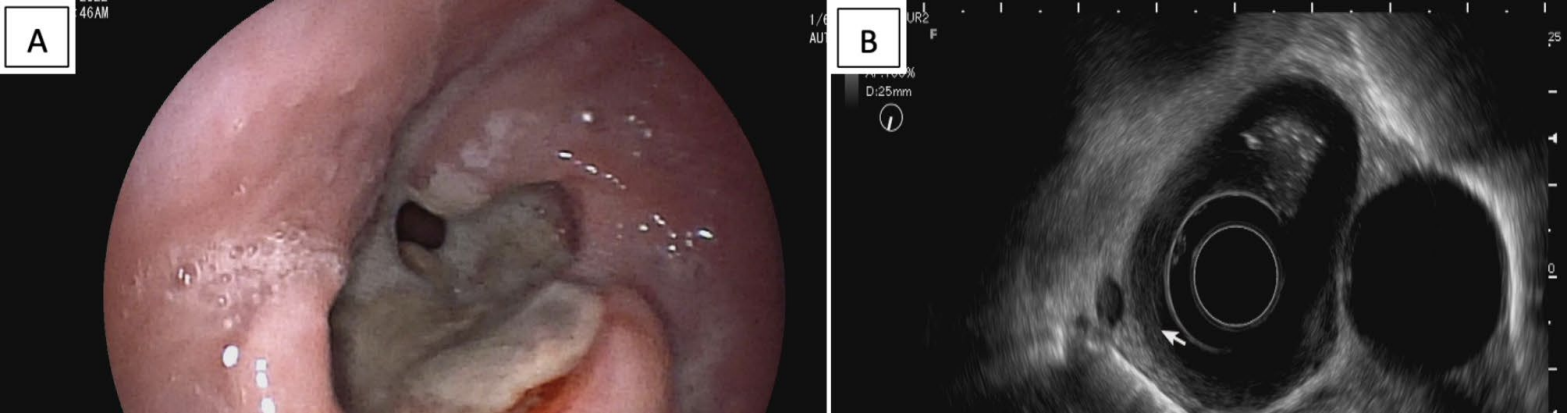
**A**: gross endoscopical view of the distal third of the esophagus, showing an ulcerative mass with raised edges and active bleeding. **B**: echoendoscopical view of the distal third of the esophagus, showing an infiltrative transmural mass, with fat spiculation

An upper digestive endoscopy (UDE) was performed, and a transmural necrotic ulcer with raised nodular edges and a deep bottom with a dark gray base, covering 80% of the circumference was observed. An echoendoscopy was performed, and it was suggestive of stage T3 esophageal neoplasia (Fig. 1).

**Fig. 2:**
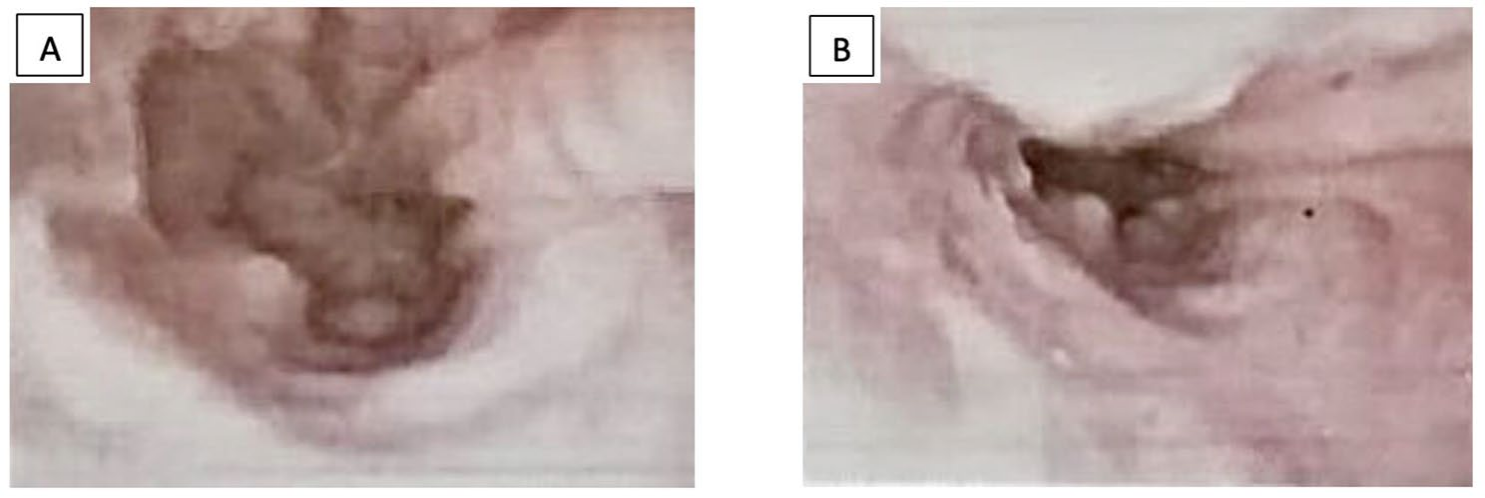
**A**: endoscopical view of the distal third of the esophagus two months since the beginning of treatment, where resolution of the lesion is seen. **B**: residual scar tissue

Pathology reports informed of ulcerated lesions with filariform bacterial colonies in the tissue compatible with esophageal actinomycosis (Fig. 2).

**Fig. 3:**
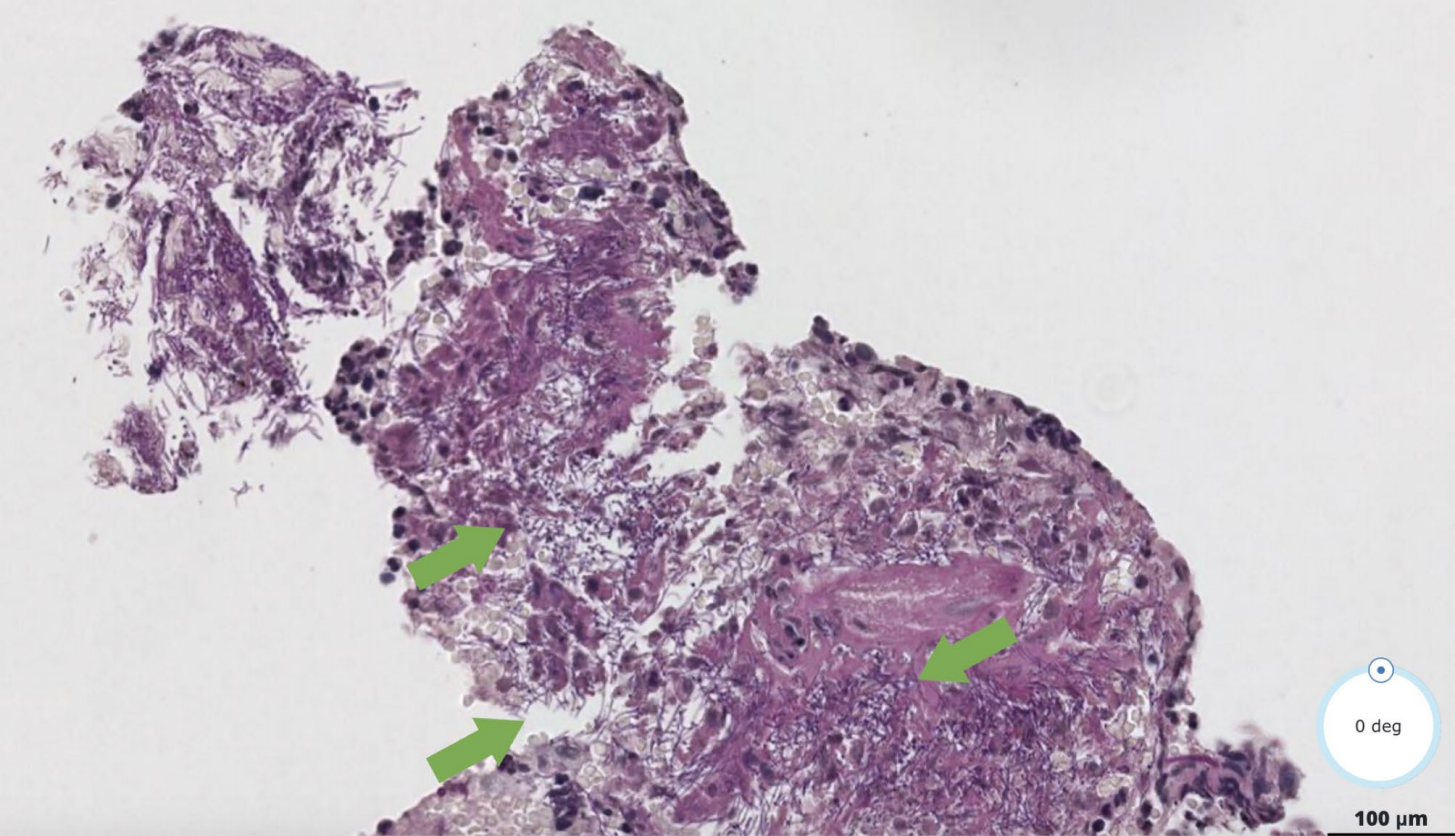
Optical microscopy 20x showing basophilic filamentous bacteria (green arrows), compatible with actinomycosis

Other infectious agents were excluded by negative staining and cultures, including tuberculosis and fungi. Immunohistochemistry was negative for cytomegalovirus. A chest tomography showed thickening of the distal esophagus, without evidence of enlarged intrathoracic lymph nodes. An abdominal tomography provided no relevant findings. HIV serologic testing was positive, with a viral load of 288 000 copies/ml and CD4 + lymphocyte count of 314 cells/ml. The patient was started on oral amoxicillin 500 mg three-times a day; and after two weeks, combined antiretroviral therapy (CART) with a daily combined fixed dose of Tenofovir-disoproxil fumarate/Lamivudine/Dolutegravir 300/300/50 mg was added. Two months later, a new UDE showed that the esophageal ulcer resolved completely, leaving only a residual scar and no relevant findings in the gastric chamber (Fig. 3), with the patient referring remarkable improvement of dysphagia and odynophagia. Similarly, the patient recovered 9 kg of weight in this time.

**Fig. 4:**
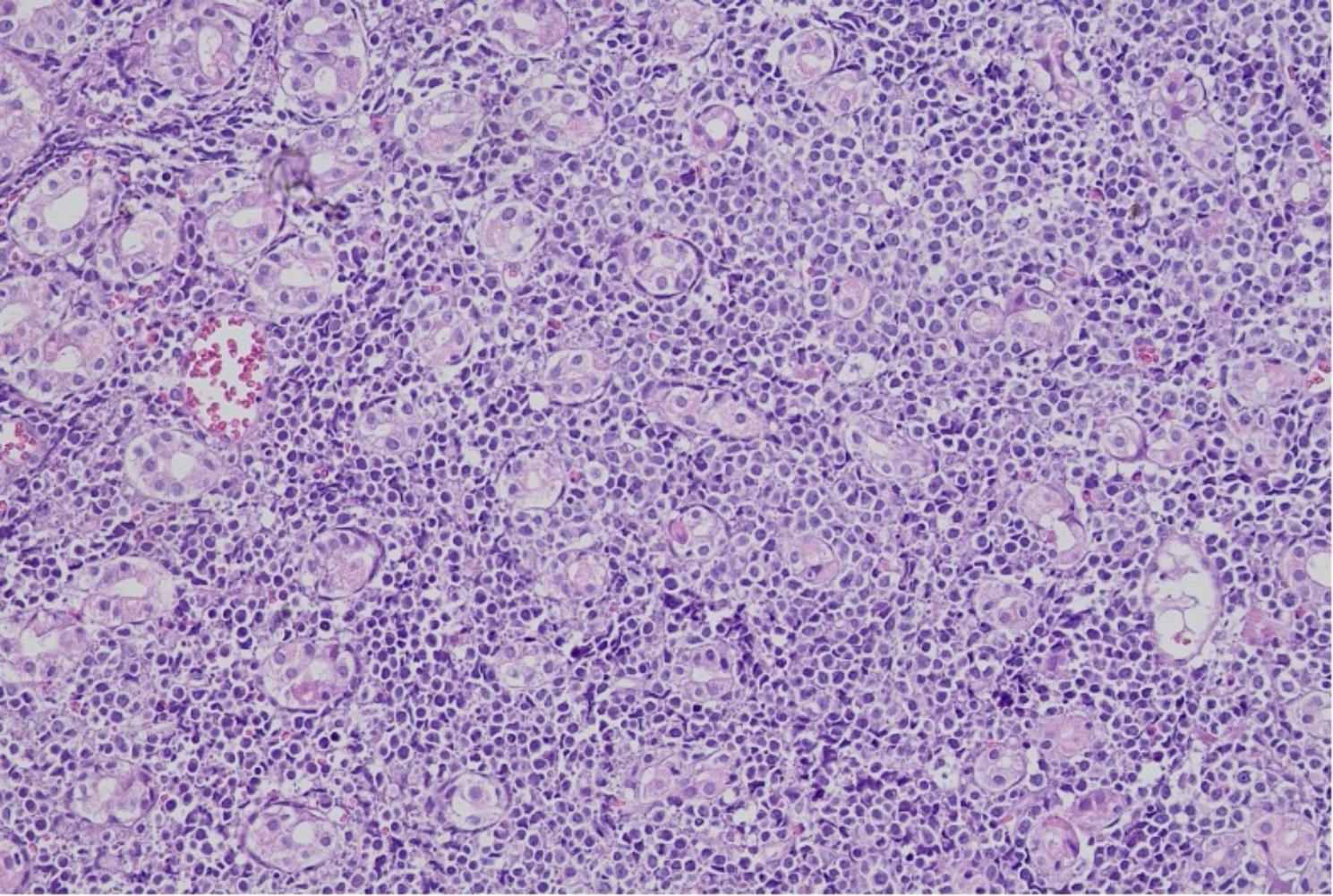
Optical microscopy 20x showing reactive follicles with neoplastic cells invading the marginal zone

Four months after the treatment started, the patient returned to the infectious disease outpatient clinic due to a new onset episode of moderate epigastric pain without fever or further symptoms. Blood biochemistry was relevant for moderate anemia (Hb 8.9 g/dl) and lymphopenia (total lymphocyte count 922 cells/mm3). He underwent a new UDE, where a residual esophageal scar was found, as well as a 30 mm ulcer in the gastric corpus, with raised edges and a dirty fibrin bed, friable to rubbing. The pathology and immunohistochemistry of this new gastric lesion were consistent with diffuse large B-cell non-Hodgkin lymphoma with germinal center (Fig. 4).

Unmasking of a previously unknown neoplasia in a patient who had recently started CART was suggestive of IRIS. Currently, the patient receives chemotherapy with the R-CHOP scheme (rituximab, cyclophosphamide, doxorubicin, vincristine and dexamethasone), prophylaxis with trimethoprim/sulfamethoxazole 160/800 mg/day, and continues taking CART and amoxicillin 1.5 g/day with a slow favorable evolution.

## Discussion and conclusion

Actinomycosis is a rare disease caused by bacteria from the genus *Actinomyces* spp., which colonizes the gastrointestinal, respiratory, and genitourinary tract as part of normal microbiota. However, chronic injury of a colonized area may lead to disease. [1, 3, 8] Cases have been described in PLWH and most reports describe lymphocyte T CD4 < 200 cell/mL. [4–7] Furthermore, reports usually highlight underlying conditions which accompany the diagnosis of esophageal actinomycosis, such as esophageal candidiasis, cytomegalovirus esophagitis or neoplasia.

Esophageal actinomycosis does not usually involve the distal third of the esophagus due to its distance from the oropharynx, [9] but can present as chronic esophageal ulceration [10, 11] or even mimicking esophageal carcinoma, [12–15] as in our case. Neurological dysphagia leading to aspiration constitutes a further risk factor that has been reported in some cases and is associated with distal esophageal compromise. [10, 11, 15] In the case we describe, probably both the digestive mucosa MALT-lymphoma-associated changes as well as HIV-associated immunosuppression led to the development of esophageal actinomycosis.

Clinical manifestations of esophageal actinomycosis range from dyspepsia and dysphagia to weight loss and wasting, mimicking the clinical presentation of a neoplasm. [16] While our patient had dysphagia, but was able to eat and drink, and had a CD4 + T lymphocyte count greater than 200 cells/ml, it is possible that the actinomycosis alone did not account for the wasting syndrome and therefore, further studies to explain emaciation were justified. Even though HIV infection could justify the weight loss, the not so low CD4 + T lymphocyte count (314 cells/ml) strongly leads us to believe that this was the result of underlying neoplasm.

Actinomycosis shows a good clinical response to long courses of amoxicillin, penicillin G, doxycycline, or metronidazole, ranging from 6 to 12 months. [7] Such is the case of this patient, whose mass resolved leaving only a well healing scar without any evidence of strictures and resolution of all initial symptoms.

This patient presented HIV-associated immunosuppression [1, 4] as the only risk factor for invasive actinomycosis. Nevertheless, it has been reported that PLWH may present actinomycosis with CD4 cell counts as high as 300 cells/mL. [6, 17, 18] Other small studies in patients with invasive actinomycosis showed that it is a surrogate marker of poor prognosis in this specific vulnerable hosts. [19] Despite our patient had an initial CD4 count of 314 cells/ml, due to immunosuppression stigma and presence of neoplasm, we considered him to be in an advanced stage of HIV.

Proper CART naive-treatment was started with the combine dose formulation of Tenofovir-DF/Lamivudine/Dolutegravir. After 12 weeks of treatment, he reached a viral load of 164 copies/ml. Even though recurrence of invasive actinomycosis is extremely rare, [20] presence of immunocompromised state such as untreated HIV infection; puts patients increased risk of recurrence especially if the CART is inconsistent. Our case did not present recurrent actinomycosis because there was clinical and structural resolution of esophageal ulcers. In the context of viral control with appearance of new symptoms, it’s reasonable to consider other opportunistic infections. The UDS showed one new gastric lesion compatible with unmasked non-Hodgkin lymphoma-associated IRIS after CART and actinomycosis treatment.

In conclusion, despite its unusual occurrence, esophageal actinomycosis in PLWH in an advanced stage should arise suspicion for further opportunistic conditions that overlap or hide the clinical manifestations. PLWH and actinomycosis should be strictly monitored until viral control is achieved, and antibiotic courses are completed.

## Data Availability

The data that support the findings of this study are openly available.

## Abbreviations

HIV: Human immunodeficiency virus
PLWH: persons living with HIV
ART: Antiretroviral therapy
R-CHOP: chemotherapy scheme based in rituximab, cyclophosphamide, doxorubicin hydrochloride (hydroxydaunorubicin), vincristine sulfate (Oncovin), and prednisone
IRIS: immune reconstitution inflammatory syndrome
Hb: hemoglobin
PCR: Polymerase chain reaction
